# Response to Comments on “Oral Tranexamic Acid Treats Papulopustular Rosacea by Improving the Skin Barrier”

**DOI:** 10.1111/jocd.70151

**Published:** 2025-04-21

**Authors:** Zining Xu, Biao Yu, Bingyang Xu, Shuhong Ye, Yuxin Qing, Bin Zhao, Sun Hong, Na Wu, Jiawen Wu

**Affiliations:** ^1^ Department of Dermatology The Second Affiliated Hospital of Xi'an Jiaotong University Xi'an China; ^2^ Department of Dermatology Taihe Hospital, Hubei University of Medicine Shi'yan China; ^3^ Department of Dermatology Xi'an International Medical Center Hospital Xi'an China; ^4^ Department of Neurology The Second Affiliated Hospital of Xi'an Jiaotong University Xi'an China; ^5^ Department of Nursing The Medicine of Xi'an Jiaotong University Xi'an China

**Keywords:** oral, rosacea, skin barrier, tranexamic acid

Dear Editor,

We appreciate the thoughtful comments and questions raised regarding our recent publication, “Oral tranexamic acid treats papulopustular rosacea by improving the skin barrier.” Below, we address the key concerns and suggestions highlighted in the correspondence:

- **Long‐term safety of oral tranexamic acid (TXA):** We acknowledge the concern regarding the long‐term safety of oral TXA, especially considering the chronic and relapsing nature of rosacea [1]. In our study, patients were monitored for 12 weeks, and we observed no significant adverse events or thrombotic complications [2]. During the last year of follow‐up, no adverse effects such as deep vein thrombosis were detected. However, we agree that long‐term studies are warranted to confirm the safety profile of oral TXA in diverse populations, particularly for extended use beyond 12 weeks. The potential association between rosacea and thrombosis further emphasizes the importance of assessing coagulation parameters in future studies.
- **Mechanisms of action: TXA's role in skin barrier restoration and inflammation modulation:** Regarding the mechanism of action, TXA likely exerts its therapeutic effects in PPR through both direct and indirect pathways. On the one hand, TXA has been shown to accelerate skin barrier recovery by promoting occludin expression. On the other hand, it exhibits anti‐inflammatory properties by modulating immune responses and suppressing angiogenesis. While our study primarily focused on clinical outcomes, further research utilizing histopathological and molecular analyses would be valuable in delineating TXA's precise effects on inflammatory pathways and skin barrier repair.
- **Generalizability of findings to broader patient populations:** Our study focused on a cohort with a median age of 28 years and a mean BMI of 20.61, which may differ from global rosacea populations that tend to have higher BMIs and an older average age [3, 4, 5, 6]. We concur that additional trials involving older and more diverse populations would enhance the generalizability of our findings and provide a more comprehensive understanding of TXA's efficacy and safety.
- **Optimizing TXA dosage: The need for standardized and dose‐responsive studies:** We agree that future studies should standardize antibiotic dosages to better isolate TXA's therapeutic effects. Exploring different dosing regimens of TXA is crucial to optimizing its therapeutic effects while minimizing potential risks. Our study utilized a fixed dose of 250 mg twice daily, which demonstrated clinical efficacy and was well tolerated. However, dose–response relationships remain unclear, and it is possible that lower doses could provide similar benefits with reduced risks, or that higher doses could enhance efficacy for patients with severe PPR. We encourage future studies to include multiple dosing arms to identify the optimal TXA regimen for rosacea treatment.
- **Efficacy of topical and localized TXA treatments:** While our study demonstrated the significant efficacy of oral TXA in improving skin barrier function and clinical outcomes [6], we recognize the potential of topical or intradermal TXA formulations to achieve similar results with potentially fewer systemic risks [7]. Comparative trials assessing oral and localized formulations would provide valuable insights into the optimal route of administration for TXA in treating rosacea.
- **Demographic and skin type considerations:** We highlighted the differential efficacy of TXA in patients with dry versus oily skin types, as classified by the Baumann system [8]. Patients with dry skin showed greater improvements in clinical erythema and skin barrier metrics, suggesting that personalized treatment strategies based on skin type may optimize outcomes. Further studies exploring these differences are essential to refine treatment protocols.
- **Differential impact on clinical erythema and papulopustular lesions:** Numerous studies have demonstrated that doxycycline can significantly reduce IGA scores in PPR. However, the persistent erythema associated with PPR remains a clinical challenge, making improvements in CEA particularly difficult. Our study observed a more pronounced improvement in the CEA score compared to the IGA score in the TXA group. It presents a compelling avenue for clinical exploration. Given these findings, TXA should be considered as part of a combination therapy in the treatment of PPR to maximize therapeutic efficacy.
- **Future directions:** Building on the results of this initial trial, we propose larger, multicenter, randomized controlled trials with extended follow‐up periods to evaluate TXA's long‐term safety, efficacy, and its impact on various patient subgroups, including those with comorbidities and different skin types. Additionally, exploring the molecular mechanisms underlying TXA's anti‐inflammatory and barrier‐enhancing effects could uncover novel therapeutic targets for rosacea management [9].
- **Addressing Demodex overgrowth and the risk of rosacea relapse:** We acknowledge the potential role of Demodex overgrowth in PPR pathogenesis and the possibility of rosacea relapse following treatment cessation. Although our study did not specifically include Demodex density assessments, it is well recognized that Demodex‐associated inflammation contributes to rosacea severity. Future studies should incorporate Demodex evaluations to determine whether TXA exerts any indirect effects on Demodex‐mediated inflammation. Additionally, investigating whether concurrent anti‐Demodex therapy could enhance the durability of TXA‐induced improvements would be a valuable avenue for research.

We appreciate the reviewer's attention to the figures in our article. Figure 1a–d were intended to provide representative clinical images of patients before and after treatment. We will work closely with the journal's editorial team to ensure that all figures are correctly included in the final published version to maintain the integrity of our study's findings.

**FIGURE 1:**
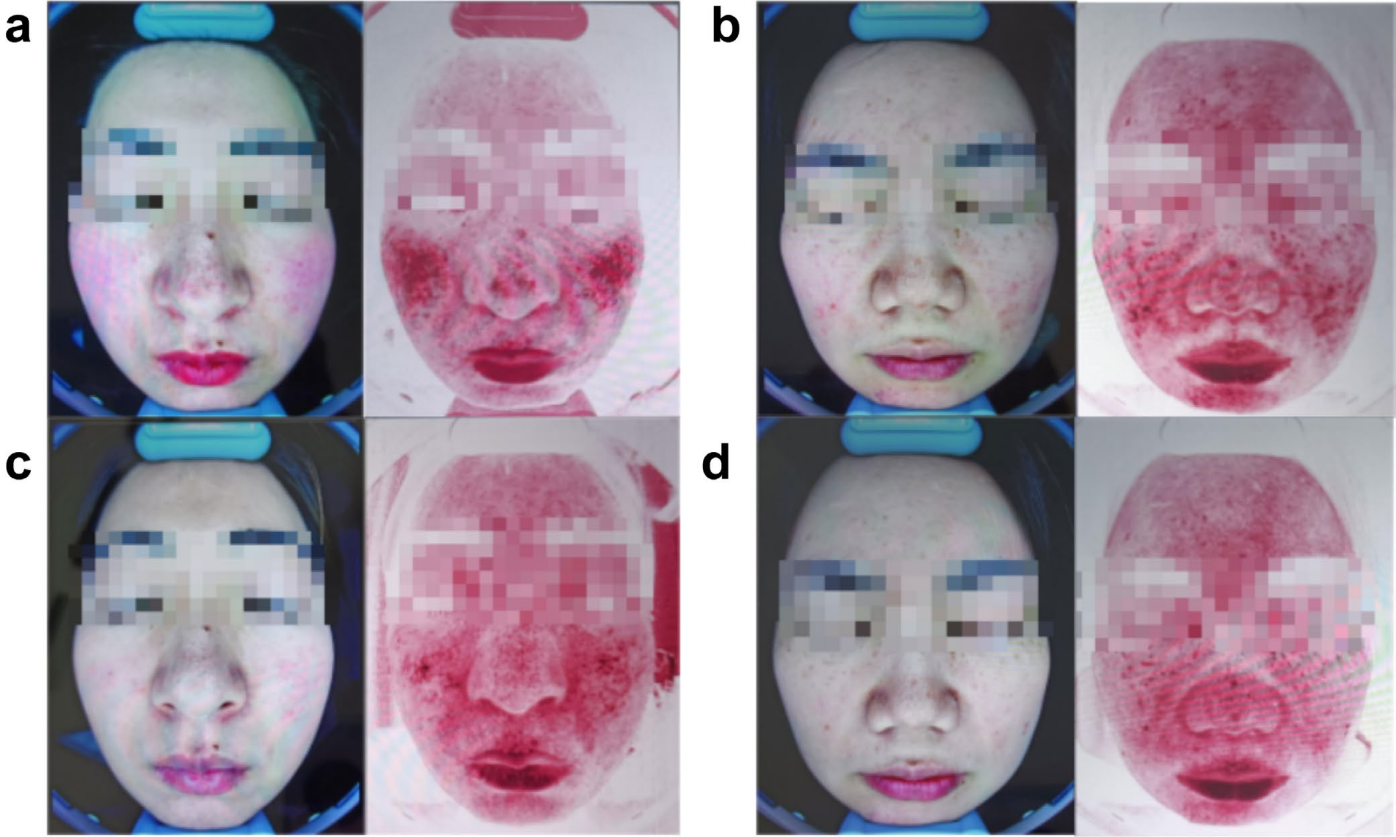
Representative images of rosacea patients at baseline and 8 weeks following traditional or TXA treatment. Pictures of the Octaspectral Facial Imager and Clinical Images at EG Baseline and at 8w of treatment (a, c). Pictures of the Octaspectral Facial Imager and Clinical Images at CG Baseline and at 8w of treatment (b, d).

In conclusion, we are grateful for the opportunity to address these important points and hope our responses provide clarity on the scope and implications of our study. We remain committed to advancing the understanding and treatment of rosacea through rigorous research.

Thank you for your consideration.

## Conflicts of Interest

The authors declare no conflicts of interest.

## Data Availability

The data that support the findings of this study are available from the corresponding author upon reasonable request.
